# Perspectives of long COVID patients on their (unmet) needs: a national quantitative and qualitative study: Marie Dauvrin

**DOI:** 10.1093/eurpub/ckac129.138

**Published:** 2022-10-25

**Authors:** M Dauvrin, L Kohn, J Detollenaere, C Primus-de Jong, C Maertens de Noordhout, D Castanares-Zapatero, I Cleemput, K Van den Heede

**Affiliations:** Belgian Health Care Knowledge Centre, Expert Health Service Research, Brussels, Belgium

## Abstract

**Introduction:**

After COVID-19, many people continue to experience various symptoms for several weeks, even after a mild acute phase, and encounter difficulties when confronted with the healthcare system. Patient associations asked the Belgian Health Care Knowledge Centre to investigate the needs of these patients to improve their management.

**Purpose of research:**

An online quantitative survey was conducted in 2021 among Belgian patients with history of COVID-19; having/had persisting symptoms for at least 4 weeks. Alongside questions on symptoms, treatment and impact on employment, Health-Related Quality of Life (HRQoL) before and after COVID-19 was measured through the EQ-5D-5L. A regression analysis identified the factors associated with the impact of long COVID on HRQoL. The qualitative approach consisted in 33 interviews and forum discussions among 101 patients.

**Results:**

1320 patients completed the online survey, most were symptomatic for more than 3 months. The average EQ-5D-5L index score was 0.85(95%CI:0.83-0.86) before and 0.65(95%CI:0.63-0.66) after infection. Duration, number and type of symptoms of long COVID significantly impacted HRQoL. More than half of the patients were unable to work. Qualitative part identified lack of empathy of health professionals, of systematic diagnostic approach, of interdisciplinary coordination. Patients felt misunderstood and developed their own diagnostic or treatment strategies. They questioned the value of medicine and resorted to non-reimbursed alternative therapies.

**Conclusions:**

Long COVID has a significant impact on HRQoL and employment. Because of long COVID, patients were confronted, sometimes for the first time, with the imperfections of the health system. Better informing the health professionals on Long COVID patterns and management options, including reimbursement possibilities, and a comprehensive interdisciplinary assessment would give them the tools to respond to the needs of these patients.

